# Investigate the bond performance of basalt FRP bars under the effect of severe conditions

**DOI:** 10.1038/s41598-025-04989-z

**Published:** 2025-06-20

**Authors:** Mohamed Heshmat, Hany El-Shafie, Fareed Elgabbas

**Affiliations:** Structural Engineering Department, Faculty of Engineering, Ain Shams University, Cairo, Egypt

**Keywords:** Basalt, FRP, Bond, Durability, Alkaline, Salts, Engineering, Materials science

## Abstract

This paper aims to evaluate the bond performance of basalt FRP bars under severe conditions such as salts, alkaline, and water. Specimens were tested under direct pull-out tensile load. The specimens were exposed to aggressive solutions at an elevated temperature of 60 °C to accelerate the degradation process. The parameters were the concrete compressive strength (CCS) (25, 45, and 60 MPa), the exposure condition (water, salts, and alkaline), and the exposure duration (30, 60, and 90 days). Seventy-two specimens were investigated in terms of bond strength, failure mechanism, and stress-slippage response. The most detrimental environment was the alkaline environment, while the salt environment had an insignificant effect on the bond strength. After 90 days of conditioning in the alkaline solution, the normalized bond strength had reduced by 17.29%, 12.74%, and 8.72% for specimens of concrete compressive strength (CCS) of 25 MPa, 45 MPa, and 60 MPa, respectively.

## Introduction

The corrosion of steel reinforcement is a major reason for the degradation of concrete structures reinforced with steel rebars, fiber-reinforced polymers (FRP) bars provide a durable remedy to the issue of corrosion, offering superior physical and mechanical properties such as high strength to weight ratio, non-corroding nature, and reasonable cost (for some types of fibers) FRPs became arousing materials for researchers to investigate and develop^1,2^. The most used fibers in the construction and strengthening process of concrete structures are carbon and glass fibers. The usage of glass fiber was preferable as it achieved the balance between being economic and having high specific strength properties. Growingly in the last decade, basalt fibers arose as a promising alternative to glass FRP materials as they have superior properties compared to glass fibers and satisfy the economic side^3^. A lot of research was performed to provide the knowledge required about basalt FRP (BFRP). However, till now the design codes and specifications do not cover this type of fibers due to lack of confidence about this material, which means more research is required to get the knowledge and confidence about the BFRP.

Benmokrane et al. investigated the physical and mechanical characteristics of BFRP bars. The results proved that BFRP bars are promising material through satisfying the requirements of the specifications for the GFRP bars^4^. Sokairge et al. investigated the creep behavior of BFRP bars, the results revealed that BFRP bars exhibited a linear relationship between e stress and the logarithm of time under different levels of stress which is similar to the behavior of the AFRP and GFRP bars^5^. Inman et al. studied the structural performance of BFRP bars as a reinforcement in concrete beams against the conventional steel rebars (CSR), as well as the environmental effects of producing the BFRP bars were studied. The BFRP bars proved their superiority through being a stronger and lighter alternative to CSR and being more eco-friendly compared to CSR^6^. Ozbulut et al. investigated the performance of BFRP bars as a repair and/or strengthening material for concrete beams using the near-surface mounted technique (NSM). Results indicated that using the NSM technique and the BFRP bars for repairing purposes can restore the original capacity of a concrete beam with corroded internal reinforcement. Moreover, the ultimate load capacity of the concrete beams can be increased using the NSM technique and the BFRP bars for strengthening purposes but at the expense of a smaller deflection capacity^7^.

Moreover, the durability is a governing property to the viability of using BFRP bars, many studies investigated the effect of severe conditions such as alkaline, acid, de-ionized water, and salt solutions on the physical and mechanical properties^8^. The alkaline environment is the most severe environment for the BFRP bars (The tensile strength retention was 88.9% after conditioning in alkaline solution for 42 days)^8^. However, the acid, salts, and de-ionized water environments have minor effects on the durability (The tensile strength retention was 93.1%, 94.4%, and 94.4% respectively, after conditioning in alkaline solution for 42 days)^8^. Altalmas et al. investigated the effect of conditioning on the bond strength of BFRP bars, the specimens were exposed to acid, ocean water, and alkaline solutions, the normalized bond strength retention was 86.1%,75%, and 75% for specimens exposed to acid, ocean water, and alkaline solutions respectively^9^. Wang et al. investigated the effect of conditioning on BFRP angles of 2.0 mm thickness. The specimens were exposed to an acidic solution with pH = 2 at an elevated temperature of 55 °C for 9 weeks. The test results showed that the tensile strength retention was 81.5%^10^. Obviously, BFRP bars have a higher resistance to an acidic solution than an alkaline solution. Therefore, most studies focused on the effect of the alkaline solution on the durability of BFRP bars. Zhao et al. investigated the durability of tensile properties of BFRP bars subjected to seawater and sea sand concrete (SWSSC) environment. The tensile strength retention of BFRP bars dropped dramatically after conditioning at 55 °C for 63 days in an alkaline solution of pH = 13.4 simulating the SWSSC. The BFRP bars lost 74% of their tensile strength^11^. Another study by Ali et al. concluded that transverse-shear strength, flexural strength, and interlaminar-shear strength for sand-coated BFRP bars reduced by 12%, 19%, and 21%, respectively, after 5000 h of conditioning in an alkaline solution at 60 °C^12^. Renyuan Qin et al. studied the long-term performance of basalt fiber reinforced composite under the effect of alkalinity and freeze-thaw cycles. The results showed that the alkaline environment along with the freeze-thaw cycles would reduce the durability of BFRC through the degradation of the resin layer^13^. Chang Su et al. made a study to evaluate the durability and long-term performance of BFRP bars using the data available from the current studies, using the existing data available Chang Su built his model to predict the strength retention of BFRP bars, and concluded that the alkaline solution would reduce the tensile and bond strength due deterioration of matrix and debonding between the resin and the fibers^14^.

The bond strength between BFRP bars and concrete as well as bond-durability are dominant properties that govern the integrity of the concrete structures reinforced with BFRP bars. El Refai et al. investigated the bond performance of the sand-coated BFRP bars when utilized in high strength concrete (50 MPa) and concluded that the behavior of the sand-coated BFRP bars was close to GFRP bars^15^. Benmokrane et al. studied the bond-durability of BFRP bars when exposed to an alkaline environment at elevated temperatures, the bond strength showed good resistance to the alkaline environment, the degradation was 16% after exposure to the alkaline environment for 6 months at an elevated temperature of 40 °C^16^. El Refai et al. investigated the bond-durability for BFRP bars and GFRP bars under several harsh environments (alkaline, salts, and acid), the results revealed that the most detrimental environments for the BFRP bars were the alkaline and salts environments, the BFRP bars lost 25% of their bond strength after conditioning at alkaline/salts solutions for 90 days at a temperature of 60 °C^9^, A. Al-Hamrani et al. investigated the bond durability of BFRP bars under the effect of sea water, sulfuric acid, and freeze-thaw cycles. The BFRP ribbed bars lost most of their bond strength 34% after exposure to sea water, while lost 20% in case of freeze-thaw and almost didn’t affect by sulfuric acid (only 5% reduction after 9 months of exposure)^17^. Lu Zhongyu et al. investigated the bond durability of BFRP bars with concrete and concrete containing fly ash, the BFRP sand-coated bars were studied under the effect of alkaline environment and seawater, and the specimens were conditioned at 40 and 60° C. Lu concluded that the bond strength decreased by 70% when conditined in alkaline solution at 60° C for 6 months in case of ordinary concrete, and the inclusion of fly ash can improve the strength retention by 10%^18^.

The need to understand the bond-durability performance of BFRP bars may be a significant obstacle to their wide acceptance in field applications. This paper presents an experimental investigation that aims to evaluate the bond-durability of BFRP bars embedded in concrete through accelerated aging tests in alkaline, salts, and water solutions at 60 °C. This study provides insight into how the BFRP bars’ bond behaves after being subject to a severe environment under varied concrete compressive strengths. The conclusions of this work shall contribute to including BFRP bars into FRP codes and specifications.

## Experimental program

The experimental program consists of two stages, the first stage aims to investigate the bond properties of the BFRP bars to be considered as a reference value, and the second stage investigates the BFRP bar’s bond properties after being exposed to severe conditions. A parametric study comparing the reference values and the value after conditioning is used to evaluate the bond durability. The parameters studied were the CCS (25, 45, and 60 MPa), the exposure duration (30, 60, and 90 days), and the conditioning environment (water, water at elevated temperature, salt solution at elevated temperature, and alkaline solution at elevated temperature). Specimens were conditioned in two different solutions (Alkaline, and Salt) simulating a specific field exposure. The experimental program consists of seventy-two pull-out specimens prepared according to ASTM D7913-14^19^, and ACI 440.3R-12^20^.

### Properties of BFRP bars

**Fig. 1:**
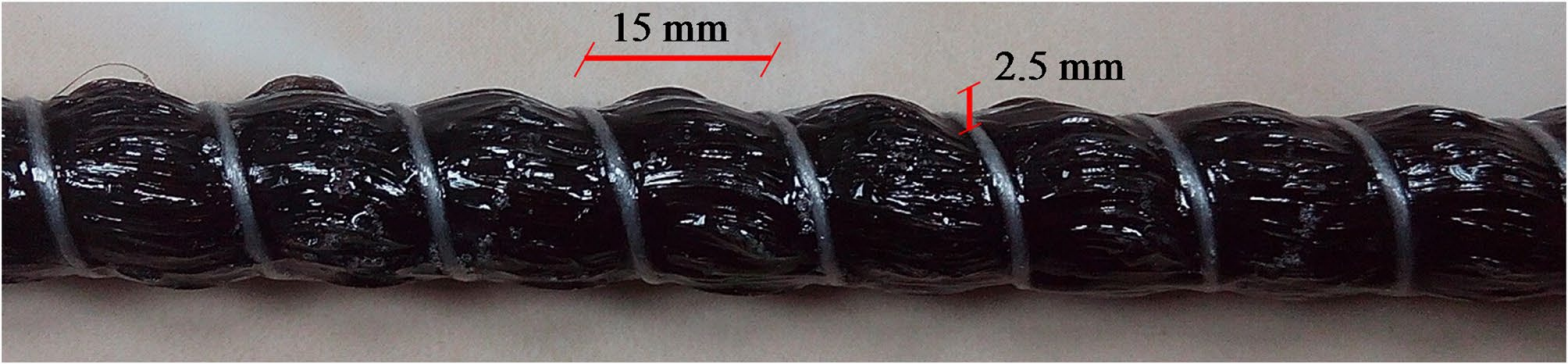
Surface configuration of BFRP Bars.

Figure 1 shows the BFRP bars used in this experimental study, which have a nominal diameter of 12 mm, with spiral ribs on a deformed surface with distinct winding filaments on the surface. Table 1 presents the mechanical tensile properties of the BFRP bars as tested by the author.

**Table 1:** The mechanical properties of BFRP bars.

Property	Diameter* (mm)	Tensile strength (MPa)	Elongation at break (%)	Modulus of elasticity (GPa)
Average	11.70	1021	2.27	45.0
SD	± 0.08	± 24	± 0.14	± 2.20

*The actual bar diameter was determined according to ASTM D7205-06 (2016)^21^, and ACI 440.3R-12^20^.

### Concrete mix

The pull-out specimens were made of normal strength concrete with target compressive strengths of 25, 45, and 60 MPa. The proportions of the concrete mix per cubic meter for the three different grades are given in Table 2. The coarse aggregates are natural crushed stones with a nominal maximum size (NMS) of 10 mm, the fine aggregates are medium natural sand, and the superplasticizer is type (F).

The specimens were cast on stages using the same concrete mixes. The CCS was determined by testing concrete cubes of 100 mm on the day of testing the pull-out specimens. The average compressive strengths are stated in Fig. 4.

**Table 2:** Constituents of the concrete mix per cubic meter.

Mix constituents	C25	C45	C60
Portland cement (kg)	350	456	500
W/C	0.57	0.45	0.35
Coarse aggregate (kg)	1117	1143	1059
Fine aggregate (kg)	745	616	706
Super-plasticizer (kg)	-----	4.56	7.50

### Environmental conditioning

The harsh accelerated aging environments adopted in this study were: (I) the alkaline solution with a pH value of 12.6 according to ACI 440.3R-12^20^, the alkaline solution simulates the concrete pore water solution. The temperature was increased to 60 °C to accelerate the reaction. The solution composed of 118.5 g of Ca (OH)_2_, 4.2 g of KOH, and 0.9 g of NaOH per one liter of water, (II) Salts solution with a pH value of 8.0 according to ASTM D1141–98 (2013)^22^, the salts solution simulates the ocean water that exists in the climate of the coastal areas. The temperature was increased to 60 °C to accelerate the reaction. The solution is composed of 24.53 g of NaCl, and 4.09 g of Na_2_SO_3_ per one liter of deionized water; (III) water at an elevated temperature of 60 °C to determine the effect of temperature on the test results. Temperature-controlled circular reservoirs were fabricated and filled with solutions. The solutions were maintained at 60 °C during the conditioning period to accelerate the reactions. The specimens were conditioned for 30, 60, and 90 days before being tested.

### Test matrix

The test matrix is presented in Table 3. Three replicate specimens were assigned to each set to guarantee the consistency of the test results. Specimens sets were labeled as follows: the solution type (W for water, S for salts, and K for alkaline), the exposure temperature (25 and 60 °C), the CCS (25, 45, and 60 MPa), the conditioning duration in days (30, 60, and 90 days). For example, specimen S60-45-60 refers to a set of three replicate specimens conditioned in the salts (S) at 60 degrees Celsius (60) with CCS of 45 MPa (45) and conditioned for 60 days (60). Concrete cubes were cast and exposed to the same conditioning as the pull-out specimens to investigate the effect of conditioning on the CCS.

### Pull-out test setup

**Fig. 2:**
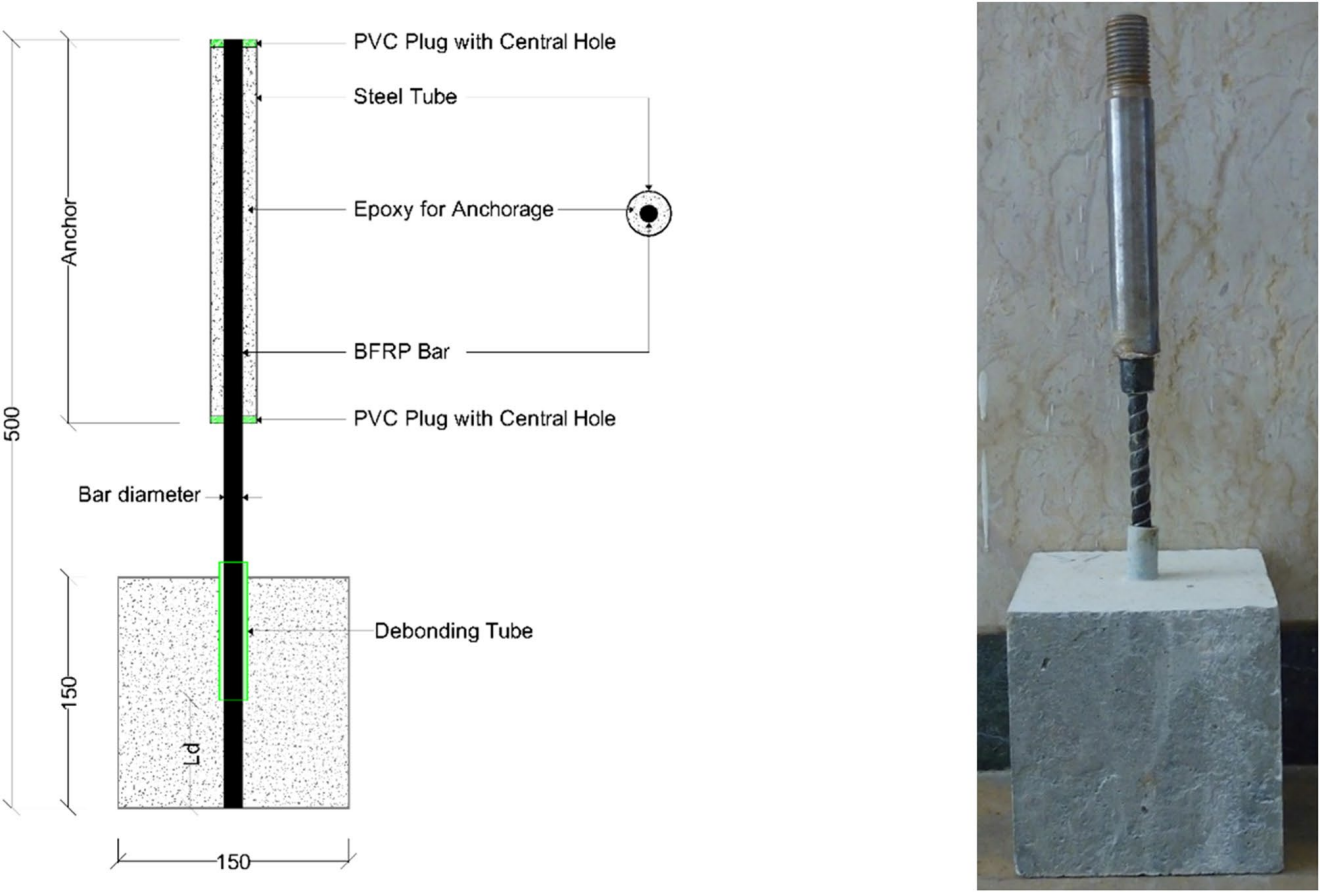
Test specimen configuration.

**Fig. 3:**
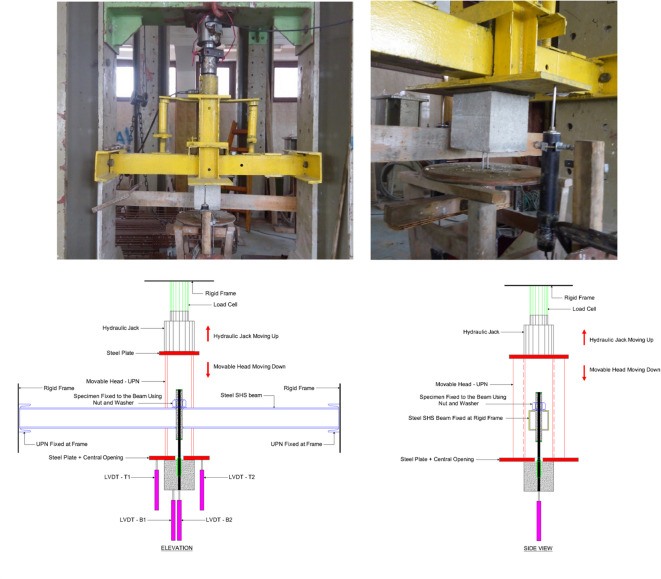
The pull-out test setup.

Pull-out tests were conducted using a load cell of 450 KN capacity. The anchorage of the BFRP bar was achieved by using a hollow steel tube of 16 mm internal diameter with a thickness of 3 mm, as shown in Fig. 2. The BFRP bar was adhered to the steel tube using epoxy resin. The test setup is shown in Fig. 3. Linear variable displacement transducers (LVDTs) were used to measure the relative displacement between the bar and the concrete cube. Two LVDTs were attached to the top surface of the concrete and two others attached at the bottom, one on the bar end and the other on the concrete. A data acquisition system (DAS) was used to record the load and slippage readings at a steady rate.

**Fig. 4:**
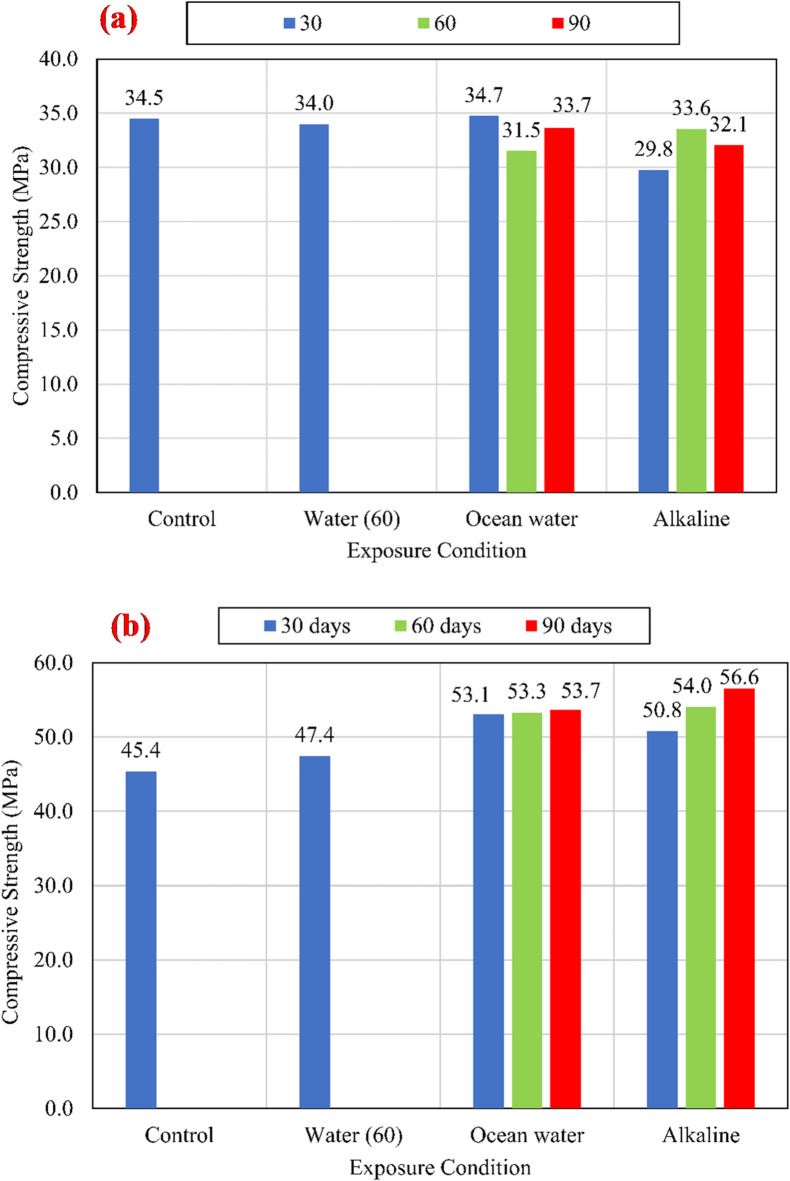

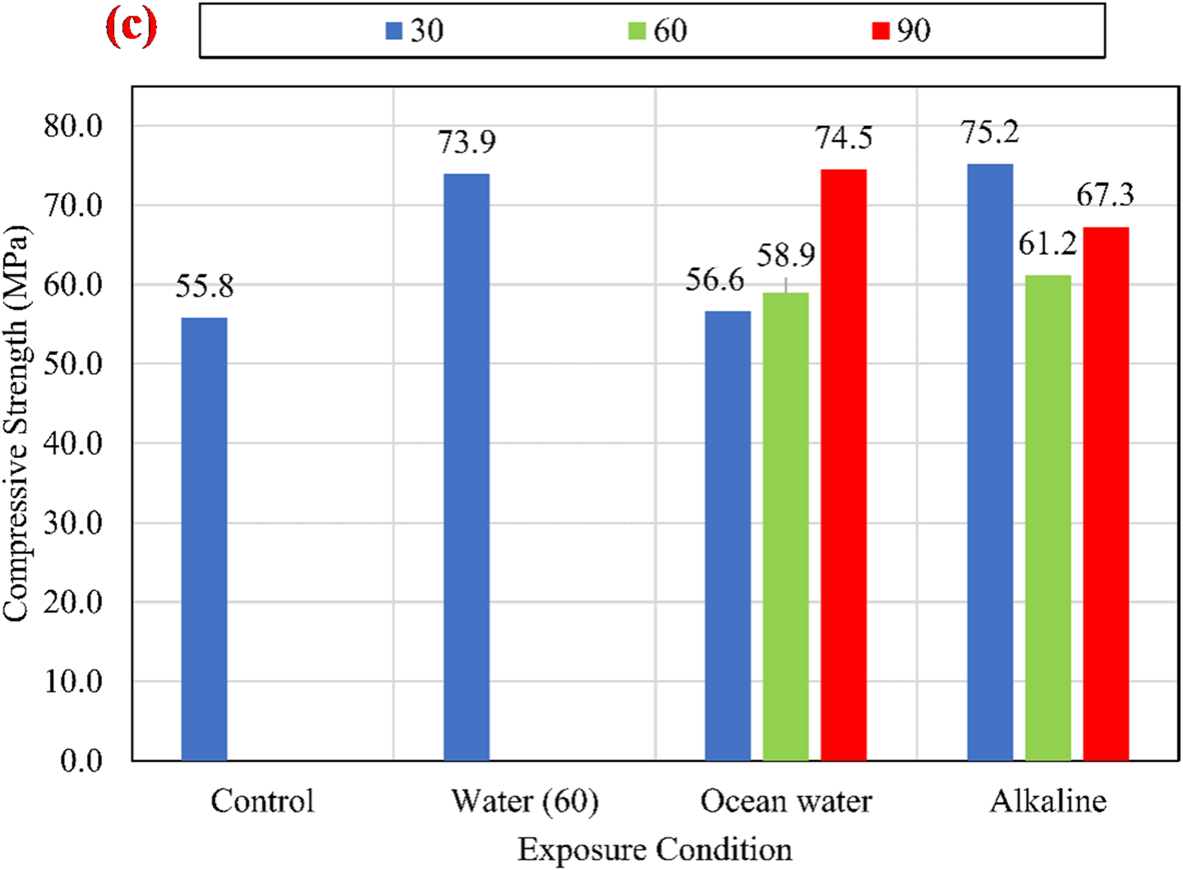
Variation of concrete compressive strength for (a) *f*_*cu*_ = 25 MPa, (b) *f*_*cu*_ = 45 MPa, and (c) *f*_*cu*_ = 60 MPa.

## Test results and discussion

The test results of 72 pull-out specimens (24 sets) are shown in Table 3 in terms of maximum bond strength, normalized bond stress, slip corresponding to max bond stress. The bond stress was calculated as follows:

1 $$\tau _{max}=\frac{{P}_{max}}{\pi{\text{ x }}d_b{\text{ x }}{L_d}}$$

Where *τ*_*max*_ is the bond strength (MPa), *P*_*max*_ is the max pull-out force (N), *d*_*b*_ is the nominal bar diameter (12 mm), and *L*_*d*_ is the bonded length (mm).

The bonded length was fixed as a multiplier of nominal bar diameter, *L*_*d*_ = 5 *d*_*b*_.

**Table 3:** Test results of BFRP pull-out test.

Specimen	τ_max_ (MPa)	C.O.V (%)	$${\raise0.7ex\hbox{${{{\tau }}_{{{\text{max}}}} }$} \!\mathord{\left/ {\vphantom {{{{\tau }}_{{{\text{max}}}} } {\sqrt {{\text{f}}_{{{\text{cu}}}} } }}}\right.\kern-\nulldelimiterspace} \!\lower0.7ex\hbox{${\sqrt {{\text{f}}_{{{\text{cu}}}} } }$}}$$	S_max, Le_ (mm)	S_max, Fe_ (mm)	τ_ons, Le_ (MPa)	τ_ons, Fe_ (MPa)
W25-25-30	25.32	9.73	4.31	3.27	1.04	0.59	1.41
W25-45-30	28.29	2.83	4.20	1.57	N/A	0.71	1.42
W25-60-30	33.42	7.47	4.48	2.19	0.68	1.22	10.27
W60-25-30	25.68	11.26	4.40	3.00	N/A	1.27	N/A
W60-45-30	29.25	2.68	4.25	1.26	N/A	1.02	1.44
W60-60-30	38.76	7.69	4.51	1.18	N/A	1.62	2.22
S60-25-30	25.10	14.66	4.26	2.76	N/A	1.16	2.44
S60-45-30	31.05	0.06	4.26	2.32	1.17	0.73	2.87
S60-60-30	34.32	15.47	4.56	2.20	N/A	1.73	3.98
S60-25-60	23.79	6.57	4.24	2.48	1.90	1.17	2.95
S60-45-60	31.51	0.42	4.32	1.72	N/A	1.10	2.20
S60-60-60	34.79	0.96	4.53	2.01	1.46	2.21	2.43
S60-25-90	24.52	6.02	4.23	2.47	1.81	0.76	2.97
S60-45-90	31.54	1.29	4.31	1.49	0.74	0.90	1.73
S60-60-90	38.88	2.11	4.51	1.42	0.64	1.27	1.88
K60-25-30	22.92	5.39	4.20	2.39	N/A	0.75	3.74
K60-45-30	30.46	7.44	4.27	2.78	2.07	1.20	11.3
K60-60-30	38.65	8.12	4.46	3.02	1.30	1.20	8.34
K60-25-60	23.73	7.92	4.10	2.15	0.57	1.10	4.53
K60-45-60	30.41	6.11	4.14	1.60	0.96	2.00	3.36
K60-60d-60	33.25	7.87	4.25	2.21	1.33	2.08	4.85
K60-25-90	20.18	3.96	3.57	1.64	N/A	0.47	2.99
K60-45-90	27.56	0.53	3.66	1.20	1.15	0.88	2.33
K60-60-90	33.51	13.72	4.09	0.84	0.36	1.05	2.44

*τ*_*max*_ = max bond stress (bond strength); *τ*_*max*_ / $\:\sqrt{{\text{f}}_{\text{cu}}}$ = normalized bond strength; *S*_*max, Le*_ = slip at loaded end corresponding to max bond stress; *S*_*max, Fe*_ = slip at unloaded (Free) end corresponding to max bond stress; *τ*_*ons*_, _*L*e_ and *τ*_*ons, Fe*_ = bond stress at the onset of slip at the loaded and free end, respectively; and N/A = data not available.

### Concrete compressive strength

Three concrete cubes for each case were cast and cured at the same conditions for the pull-out specimens to determine the effect of the elevated temperature and conditioning solution on the CCS. The concrete cubes were cast on several stages according to the order of pouring the pull-out specimens. Figure 4 shows the variation of the CCS. The differences between values reveal that the alkaline solution, salts solution, and elevated temperature had a negligible effect on the CCS in the case of *f*_*cu*_ = 25 MPa. In the case of *f*_*cu*_ = 45 MPa, the alkaline and salt increased the CCS slightly at 30, 60, and 90 days. In the case of *f*_*cu*_ = 60 MPa, the hot water has a significant effect on the CCS at 30 days. Moreover, the salt and alkaline increased the CCS after 90 days compared to the unconditioned specimens.

### Bond stress–slip response

**Fig. 5:**
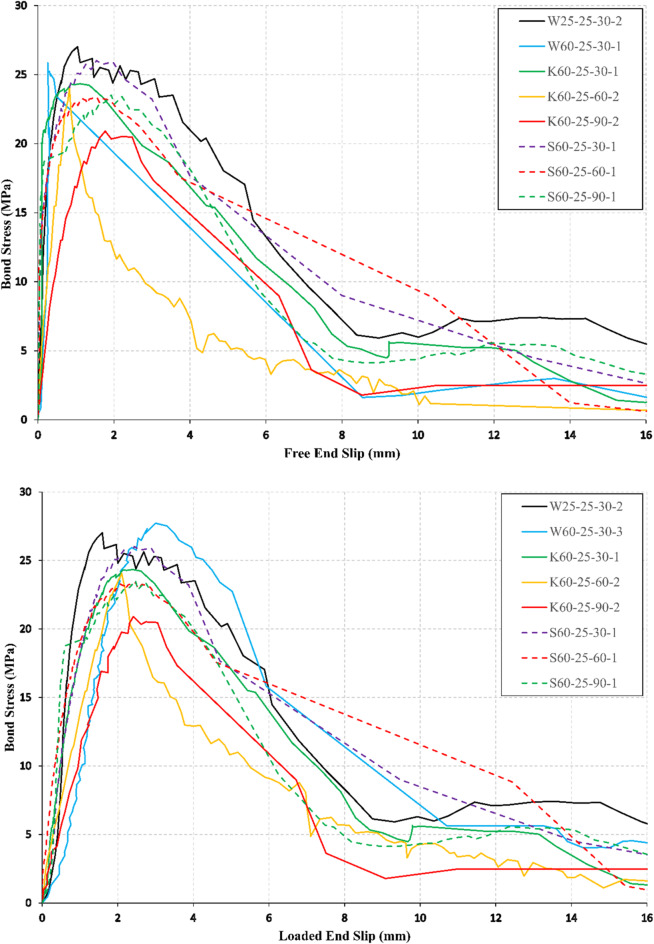
Bond stress–slip curves at free and loaded ends for *f*_*cu*_ = 25 MPa.

**Fig. 6:**
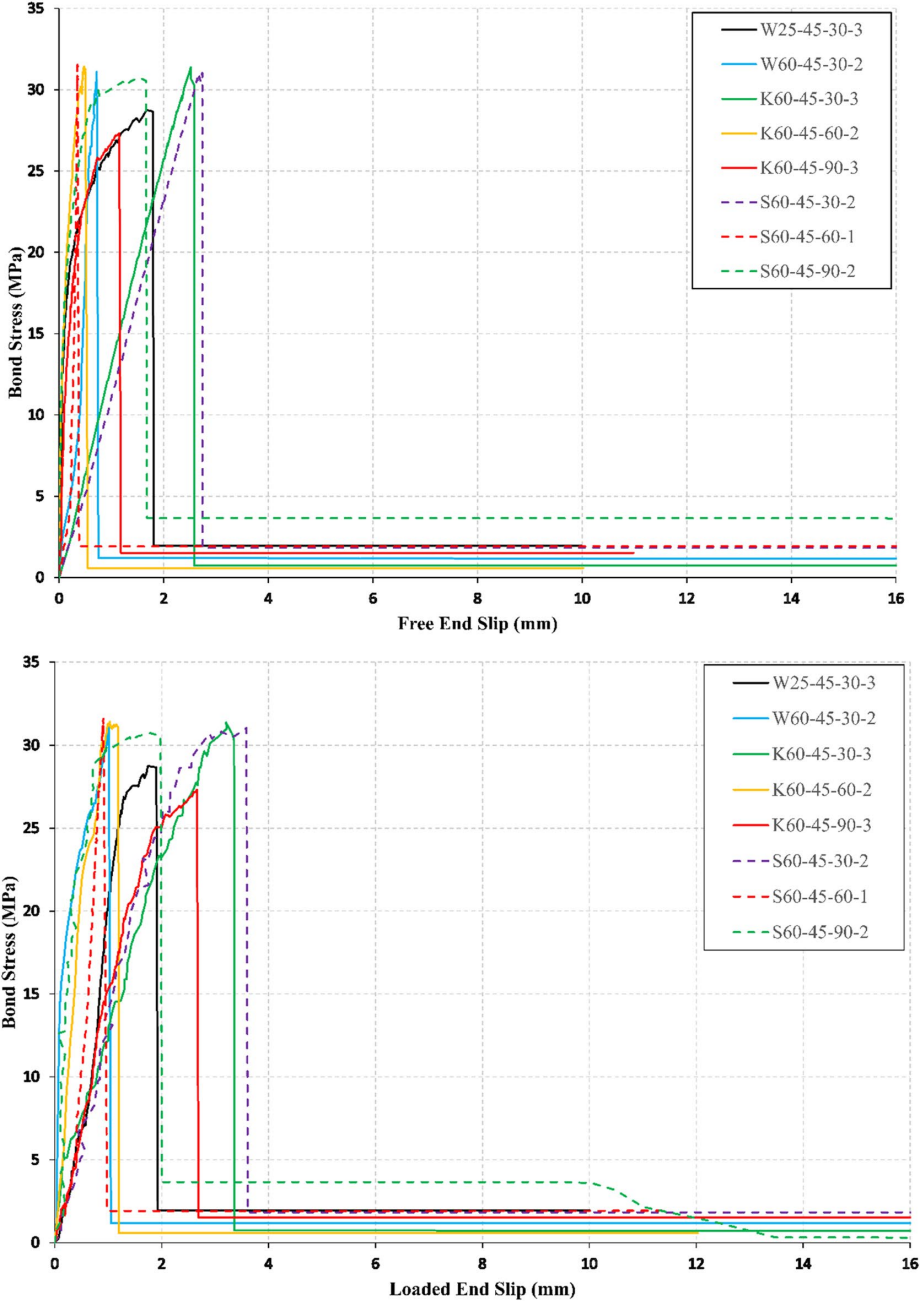
Bond stress–slip curves at free and loaded ends for *f*_*cu*_ = 45 MPa.

**Fig. 7:**
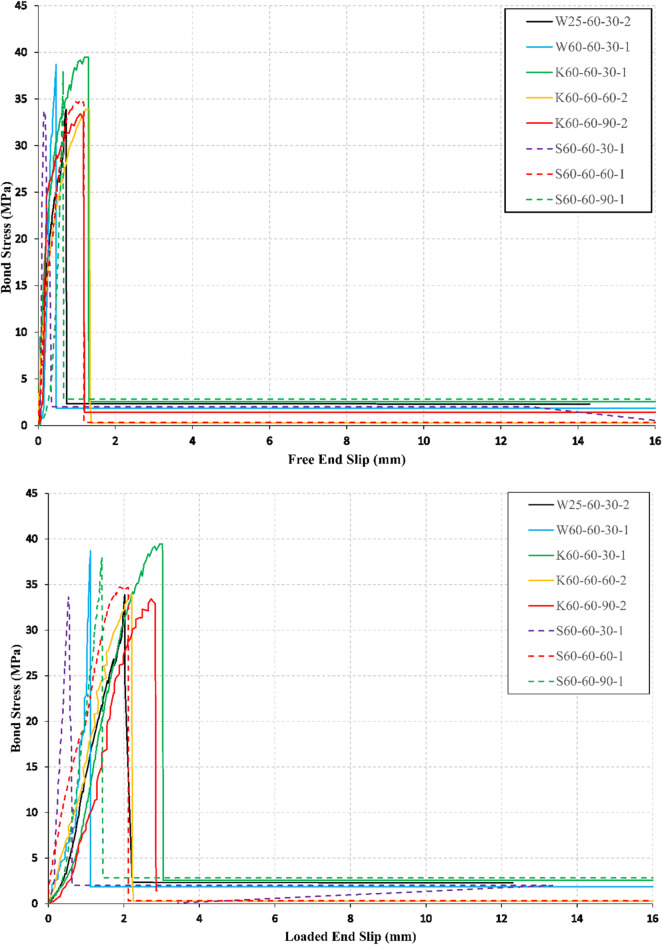
Bond stress–slip curves at free and loaded ends for *f*_*cu*_ = 60 MPa.

Bond stress-end slip relations at the free and loaded ends are illustrated in Figs. 5, 6, and 7 for all conditioned specimens. The slip of the bars was determined using the relative displacement between two LVDTs, one LVDT that records the bar displacement and the other LVDT records the concrete cube displacement as shown in Fig. 4. The ascending branch of the bond stress-end slip curves showed a linear behavior for all the specimens with a larger value of end slip at the loaded end than in the free end.

The descending branch of the bond stress-end slip curves showed a gradual decrease in the bond stress accompanied with large slip values for most of the specimens in the case of *f*_*cu*_ = 25 MPa as shown in Fig. 5, and showed a sharp decrease in the bond stress accompanied with small slip values for most of the specimens in case of *f*_*cu*_ = 45 MPa, and *f*_*cu*_ = 60 MPa, as shown in Figs. 6, and  7, respectively. The decrease in the bond stress conforms with the modes of failure observed during the test of BFRP specimens (interlaminar shear of bar layers, delamination of BFRP bar ribs, and splitting of the concrete cube). The conditioned specimens showed similar behavior to the unconditioned specimens of the same CCS.

### Bond failure mechanism

**Fig. 8:**
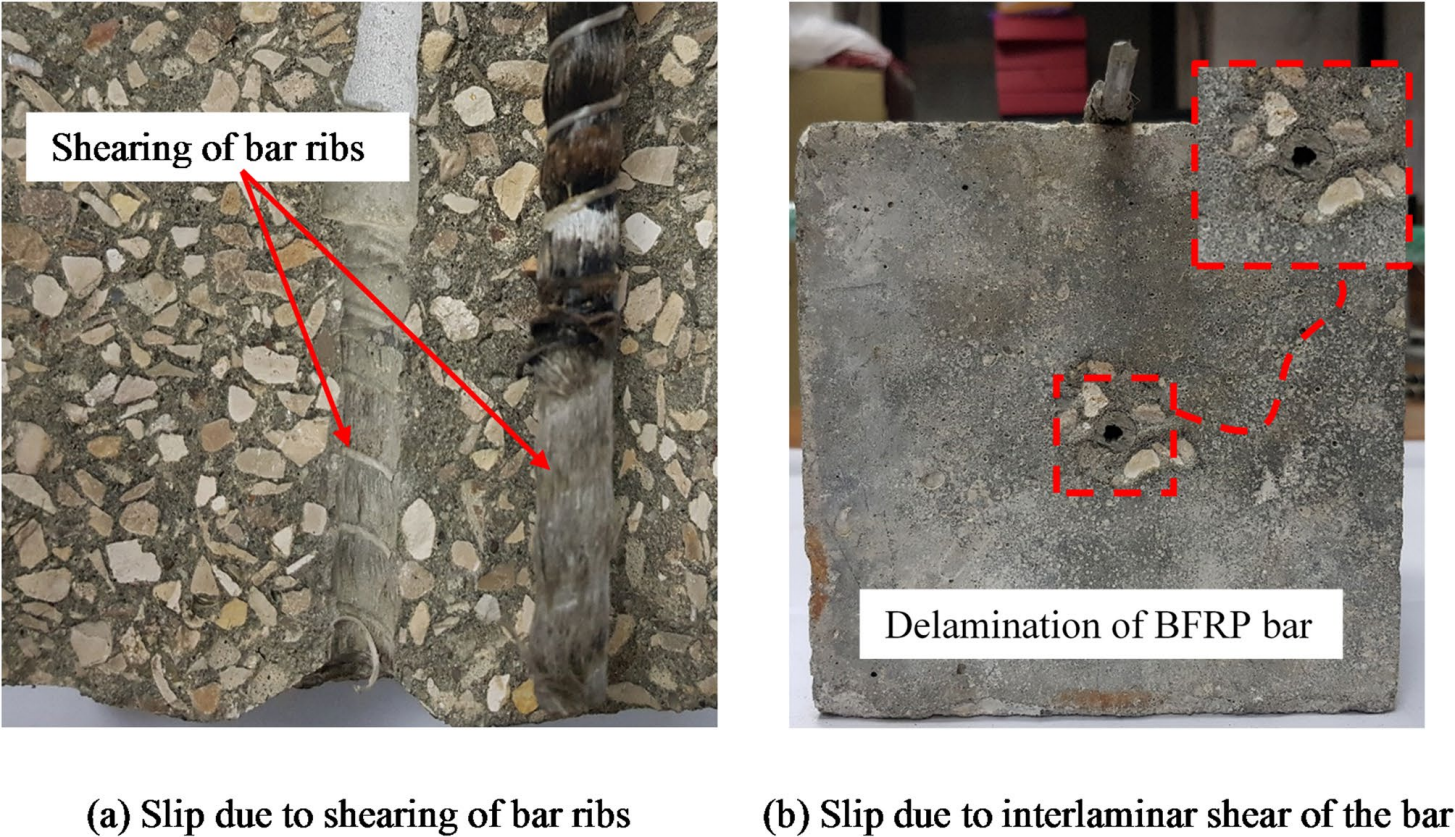
The typical modes of failure for slippage of FRP bars.

The bond strength between FRP bars and concrete consists of three components: (I) Adhesion stresses; (II) Friction stresses; and (III) Bearing stresses in case of deformed bars^23^. Belarabi and wang^24^ established three mechanisms for the deterioration of the bond between FRP bars and concrete as follows: (I) Formation of microcracks at the interface between FRP bars and concrete resulted from the expansion of solutions in the micro-voids; (II) The FRP bars and concrete have a different coefficient of thermal expansion, consequently when subjected to thermal conditioning stresses will form at the concrete-FRP interface; and (III) The degradation of the FRP bar itself resulted from conditioning (especially at the surface)^24^. These three mechanisms function together to eliminate the bond forces which lead to bond failure. In the experimental study, two modes of bond failure observed (I) Splitting of the concrete cube; and (II) Slip of the bar, which can be divided into two cases: (a) Slip due to shearing of bar ribs (Fig. 8-a); and (b) Slip due to interlaminar shear between the bar layers (Fig. 8-b).

Clear traces of the resin and basalt fiber were observed on the surface of the concrete in the embedded zone, as shown in Fig. 8a. The observed bond-slip relationships were consistent with the failure mechanism, where the degradation of the bar itself was the dominant factor in the degradation of the bond.

### Bond strength variation in conditioned specimens

#### Bond strength

**Fig. 9:**
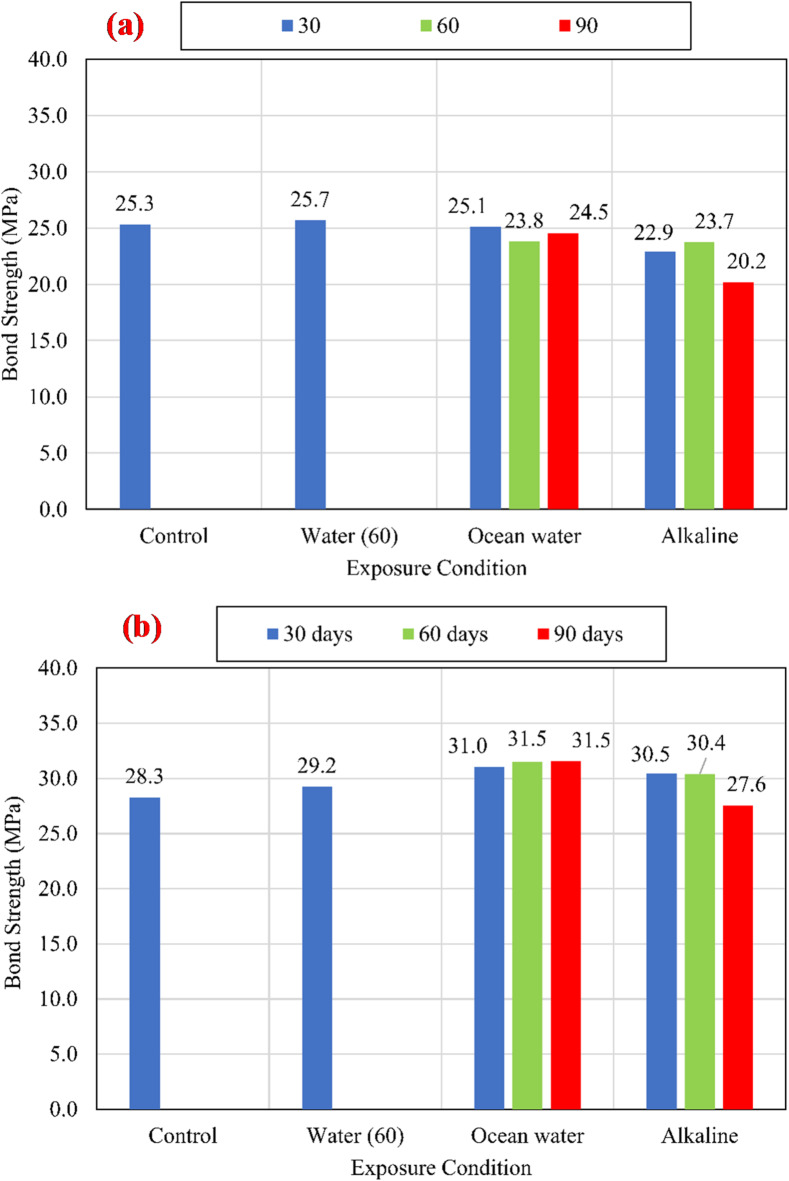

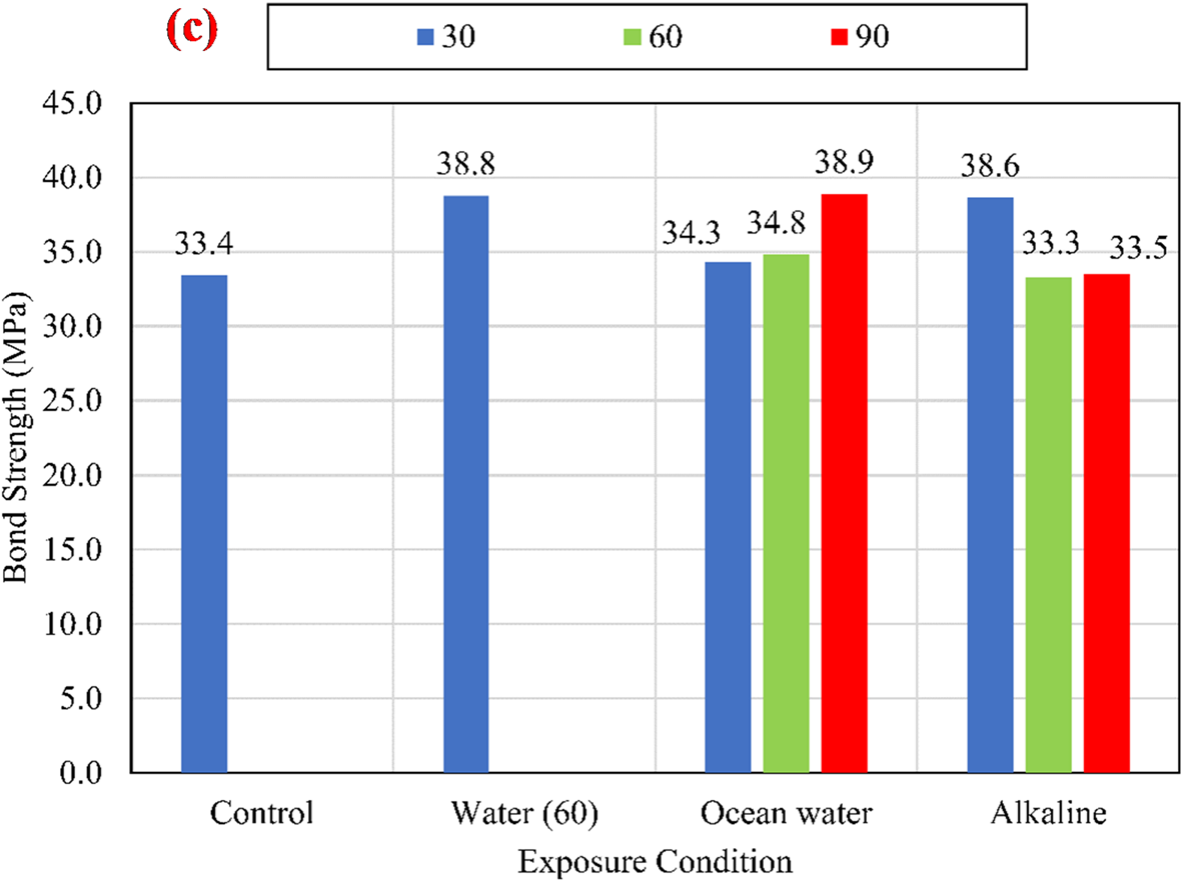
The average bond strength for the control and conditioned specimens for (a) *f*_*cu*_ = 25 MPa, (b) *f*_*cu*_ = 45 MPa, and (c) *f*_*cu*_ = 60 MPa.

**Fig. 10:**
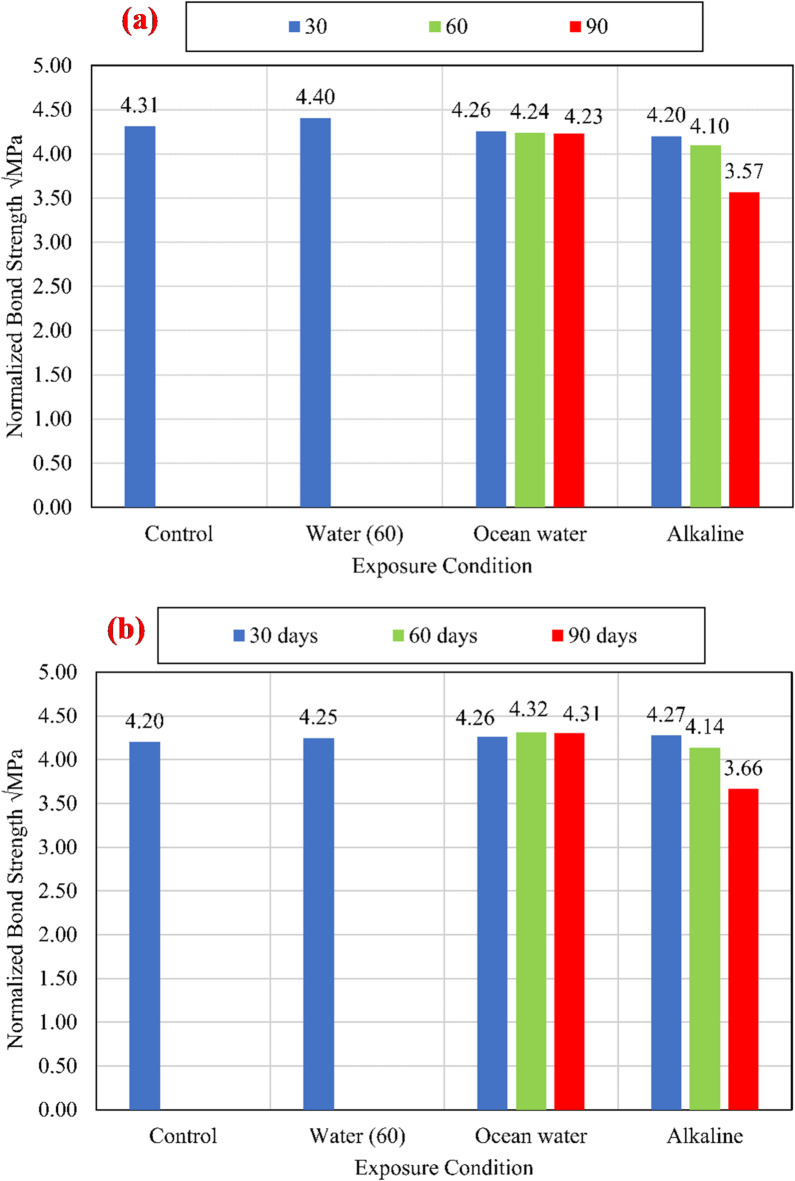

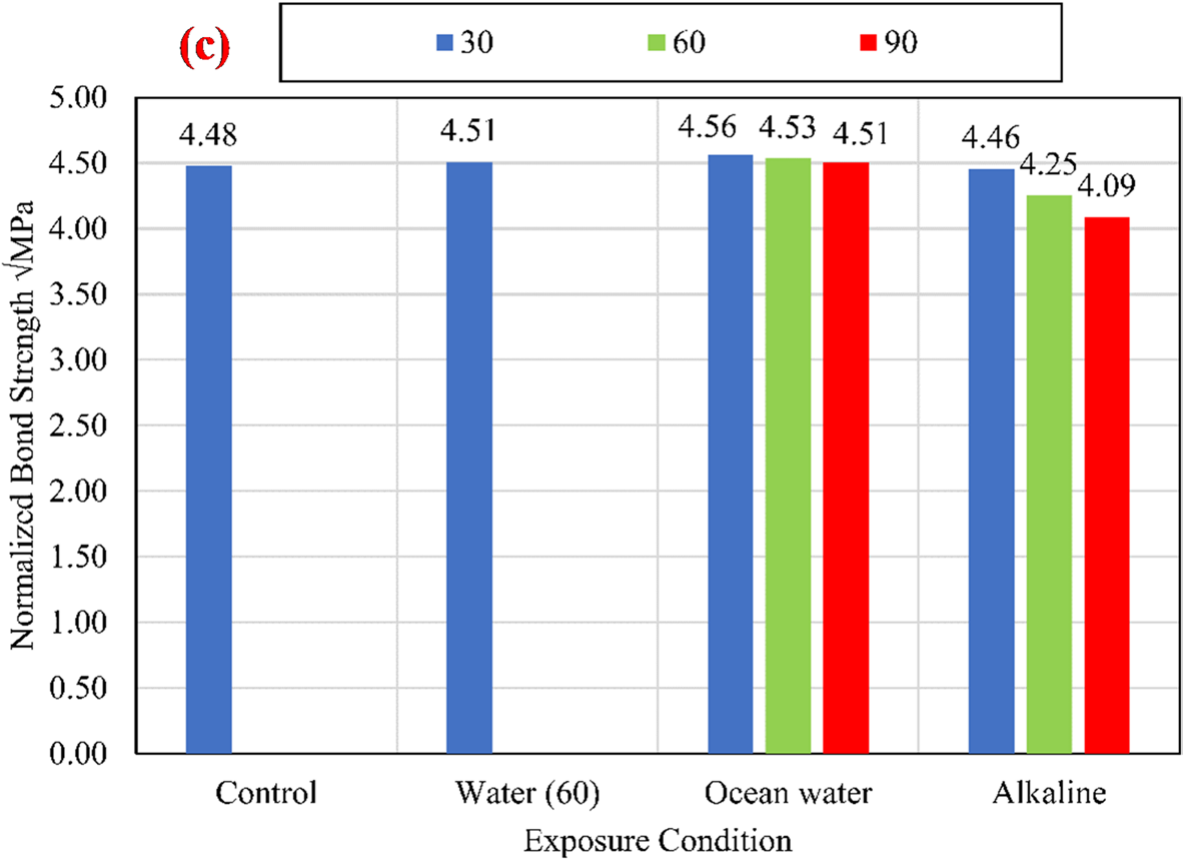
The average normalized bond strength for the control and conditioned specimens for (a) *f*_*cu*_ = 25 MPa, (b) *f*_*cu*_ = 45 MPa, and (c) *f*_*cu*_ = 60 MPa.

The charts shown in Fig. 9 compare the average bond strengths listed in Table 3 of both unconditioned (control) and conditioned specimens for different compressive strengths of 25 MPa, 45 MPa, and 60 MPa. To eliminate the effect of variation of CCS on the test results, normalized values were determined with respect to the square root of the CCS by dividing the bond strength by the square root of the CCS^9,25^, as shown in Fig. 10.

**Fig. 11:**
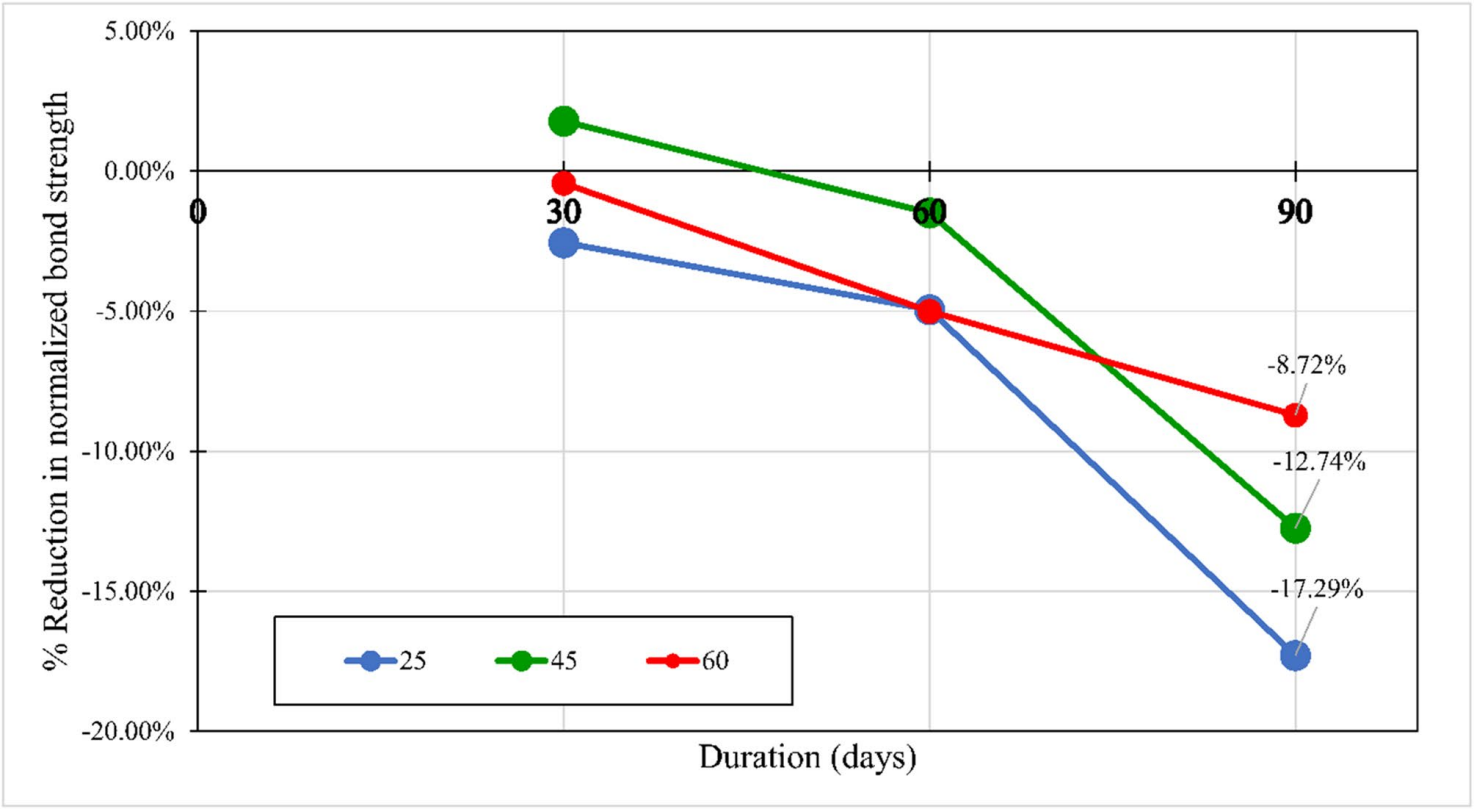
The percentage reduction in the normalized bond strength at exposure to the alkaline solution.

For all investigated CCS, test results revealed that there is no significant effect of the water at an elevated temperature of 60 °C (W60) and the salts solution at an elevated temperature of 60 °C (S60) on the bond strength compared with the control specimens. While the alkaline solution has a significant effect on the bond strength, as shown in Fig. 11. After 90 days of exposure to the alkaline solution at 60 °C, the normalized bond strengths have been reduced by 17.29%, 12.74%, and 8.72% for CCS of *f*_*cu*_ = 25 MPa *f*_*cu*_ = 45 MPa, and *f*_*cu*_ = 60 MPa, respectively, compared to the bond strength of the control specimens. The degradation of the bond was raised from the reactivity between the alkaline solution and the BFRP bar surface. Thus, for the lowest CCS of 25 MPa, the reduction was the most as it is the most permeable concrete. Therefore, as the permeability of the concrete (the lower the CCS) increased, the rate of the bond strength loss increased.

#### Slip and stiffness of the BFRP bar

**Fig. 12:**
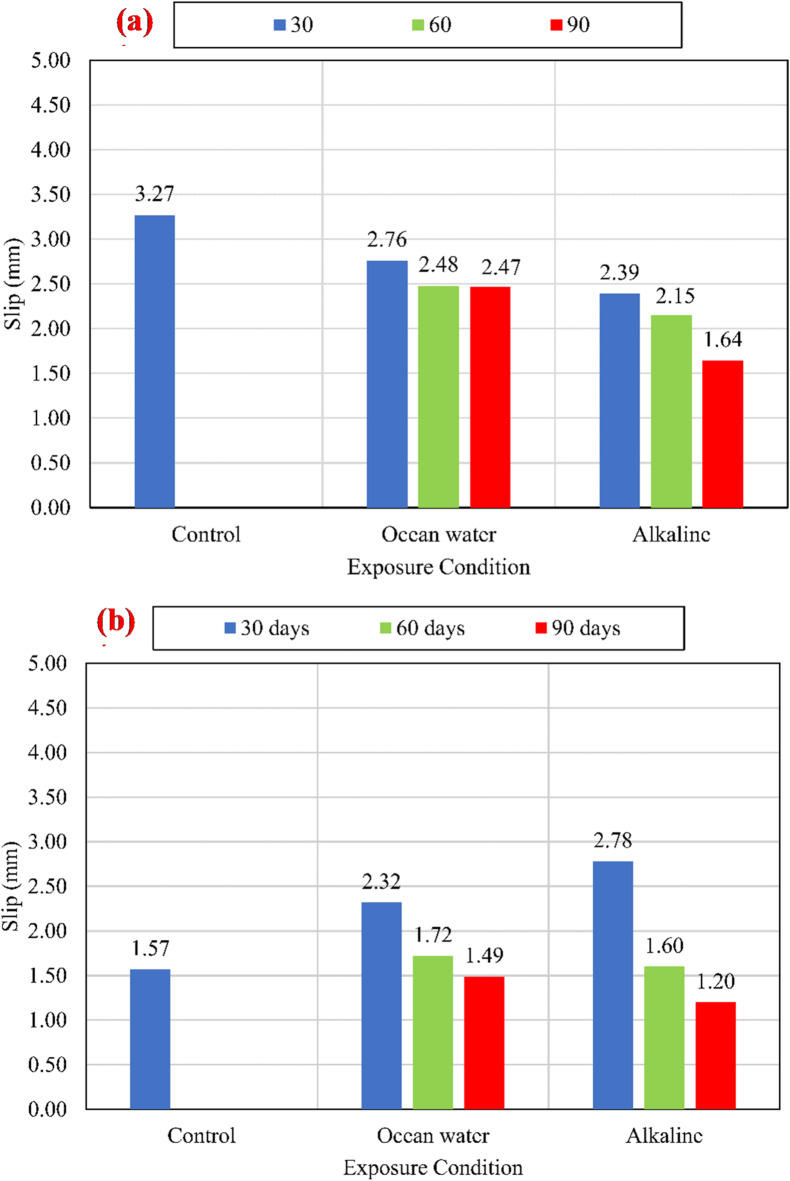

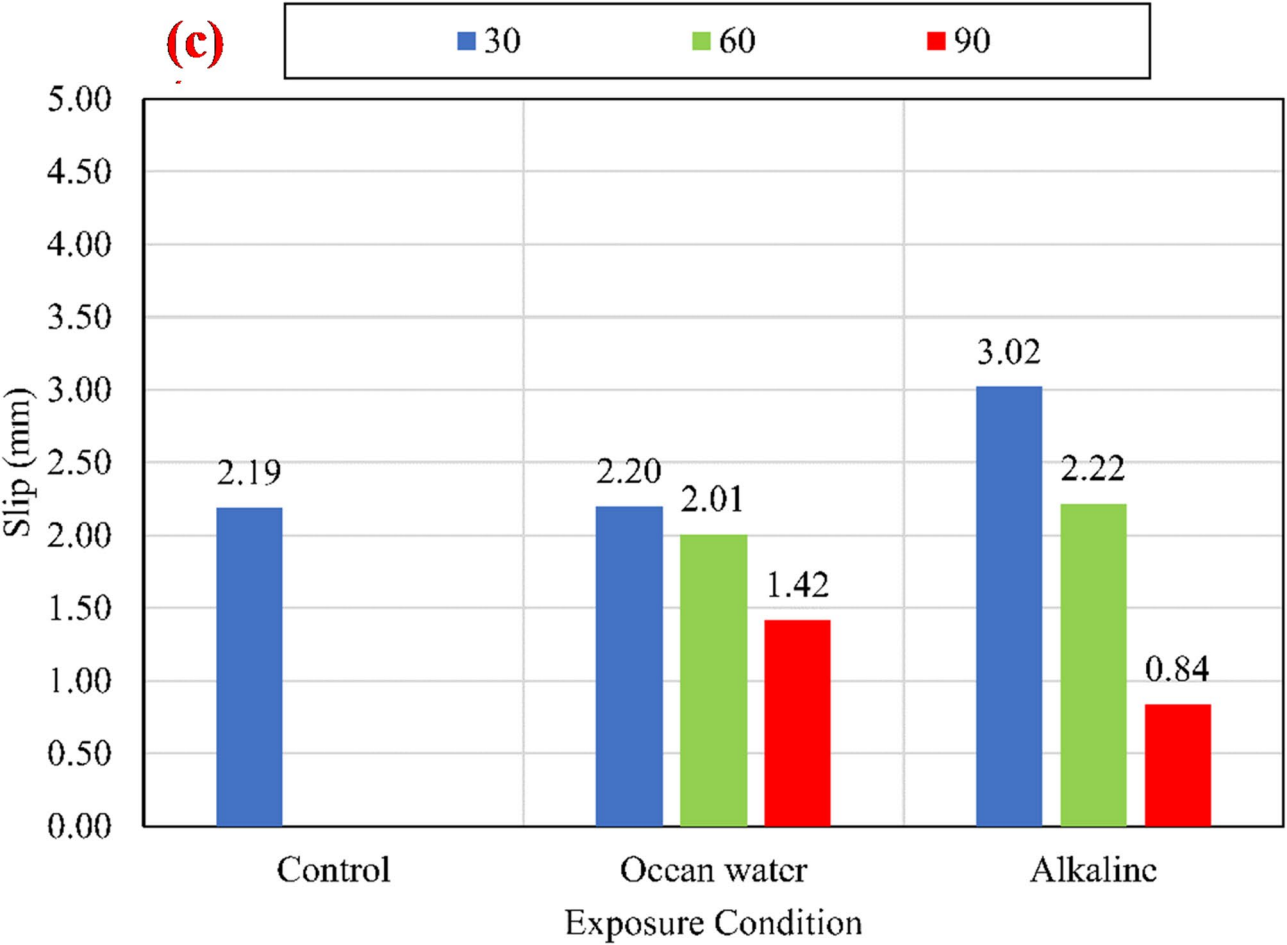
Slip at maximum stress at the loaded end for (a) *f*_*cu*_ = 25 MPa, (b) *f*_*cu*_ = 45 MPa, and (c) *f*_*cu*_ = 60 MPa.

Figure 12 shows the average slip measured at the loaded ends at the maximum stress. For CCS of 25 MPa, a continuous reduction in slip was observed after exposure to the alkaline and salts solutions. The slippage values were reduced by 26.91%, and 15.60% after exposure for 30 days in alkaline and salts solutions, respectively, compared to the slippage of unconditioned specimens. Moreover, the slippage values were reduced by 49.58%, and 24.64% after exposure for 90 days in alkaline and salts solutions, respectively. For CCS of 45 MPa, all specimens that were conditioned in alkaline and salts solutions for 30 days suffered an increase in the slip of 77.07% and 47.77%, respectively, compared to the slip of unconditioned specimens. While the slip for specimens that were conditioned in alkaline and salts solutions for 90 days were reduced by 23.57% and 5.10%, respectively. For CCS of 60 MPa, all specimens that were conditioned in alkaline solution for 30 days suffered an increase in the slip of 37.90%, compared to the slip of unconditioned specimens. While the specimens conditioned at salts solution had an insignificant increase in slip after conditioning for 30 days. After conditioning for 90 days at alkaline and salts solutions all specimens suffered a decrease in slip by 61.64% and 35.16%, respectively. The continuous variation in slip measurements with the bond strength indicates that the bond stiffness had been varied after being conditioned in the alkaline and salts solutions.

#### Adhesion stresses

**Fig. 13:**
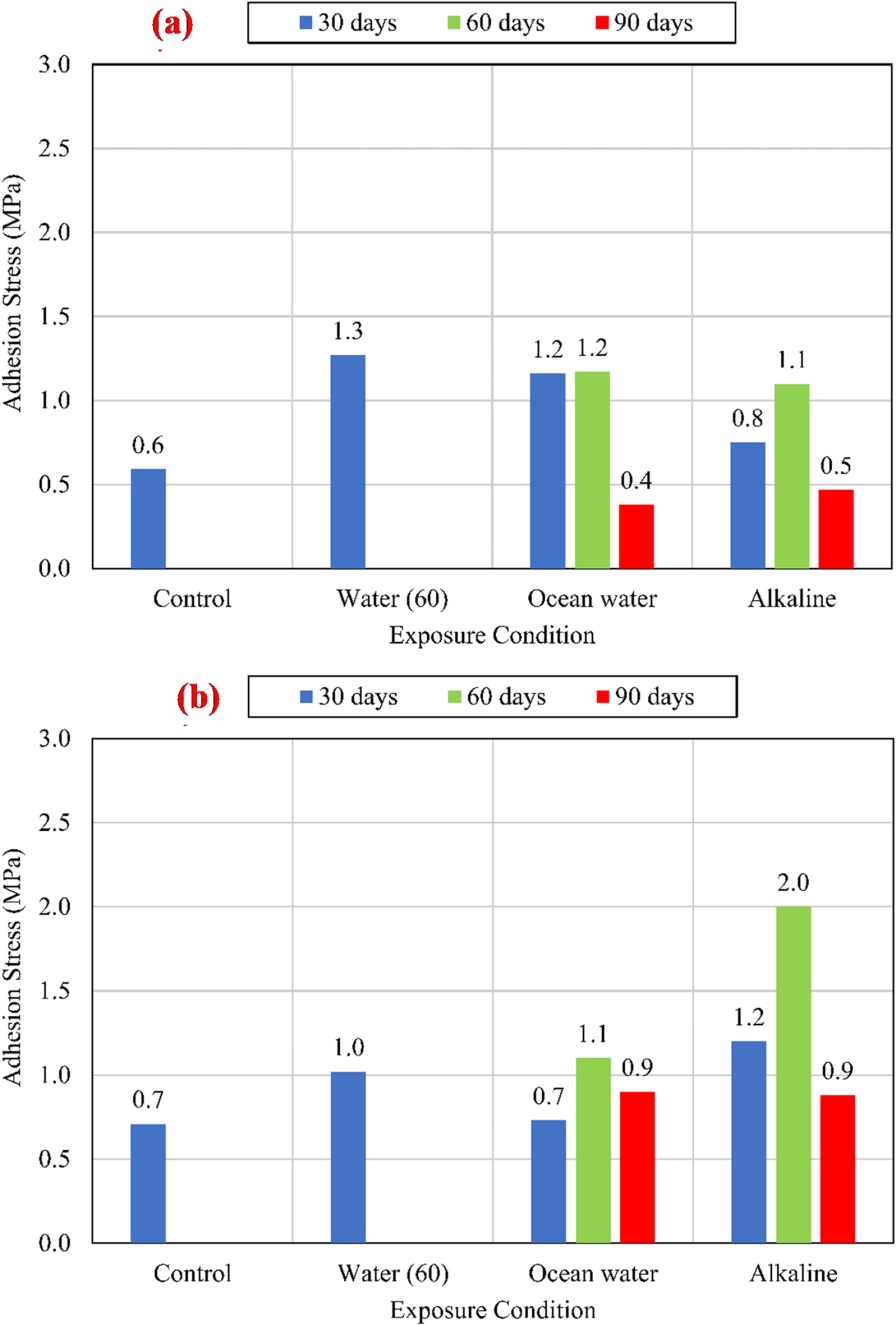

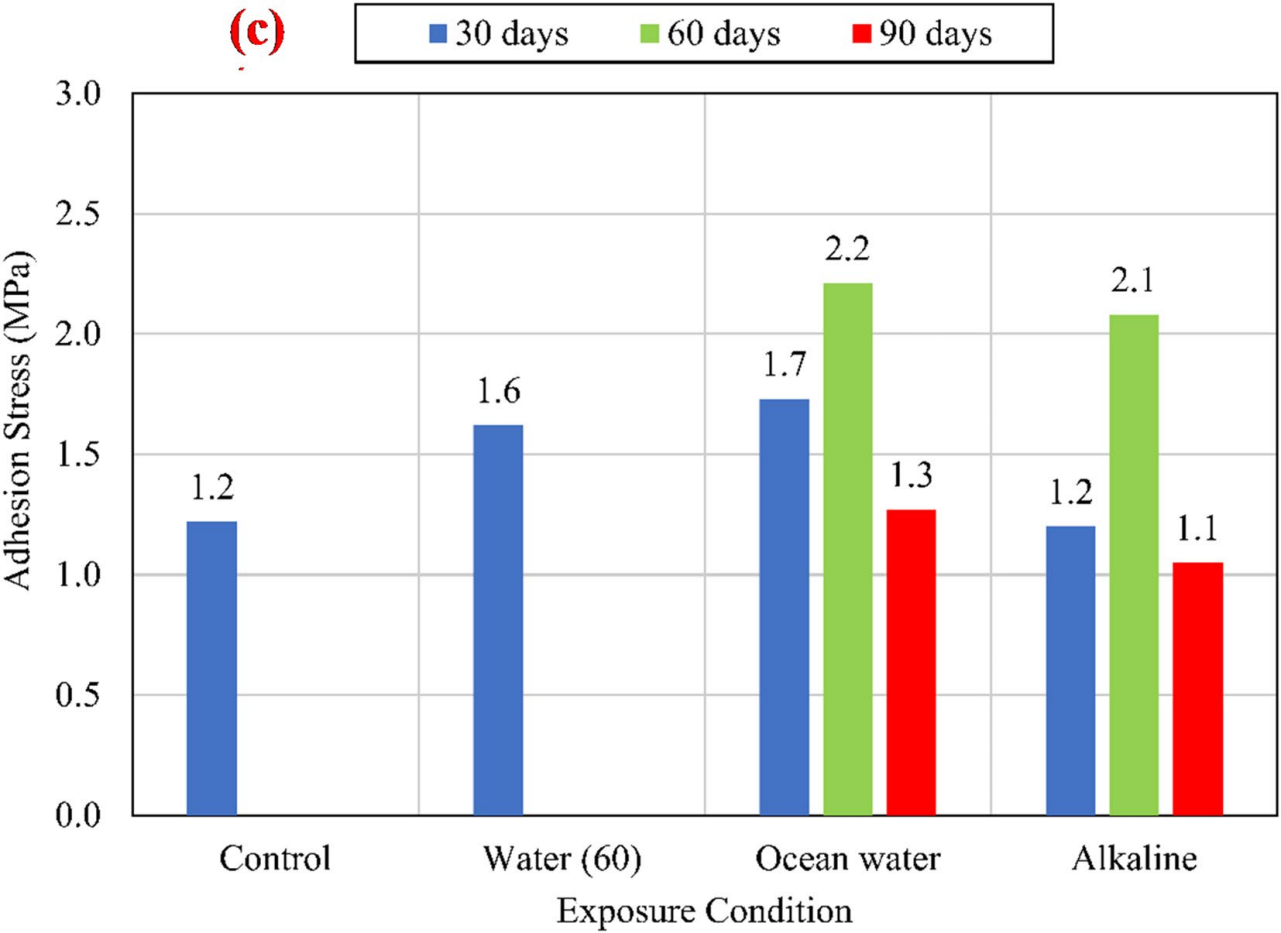
Adhesion stresses at the loaded end for (a) *f*_*cu*_ = 25 MPa, (b) fcu = 45 MPa, and (c) *f*_*cu*_ = 60 MPa.

Adhesion strength (onset stress) is the max stress that can be borne by the interface between the FRP bar and the surrounding concrete without detaching^9^, which is responsible for the behavior of bond at the early stages of loading. Figure 13 describes the onset stresses for both control and conditioned specimens for the three CCS. The onset stress was specified from the bond-slip curves at the point where the ascending branch slope changed acutely indicating the failure of adhesion strength between the concrete and BFRP bar. For all CCS, an increase of onset stress was noticed after conditioning for 30 and 60 days. On contrary, the onset stress was decreased after conditioning for 90 days. The initial increase at the early stages of conditioning can be demonstrated by two factors: (I) Swelling of the bars due to absorption of bars to the alkaline or salts solution; and/or (II) Increase of the CCS at this stage. For the long-time conditioning case (90 days), the rate of absorption decreases, due to the saturation of bars, leading to a decrease in the rate of the bar swelling, as well as the degradation of the BFRP bar surface counteracting the swelling effect resulting in deterioration of BFRP bar adhesion to concrete. The most affected CCS was at *f*_*cu*_ = 25 MPa which reduced by 33.33% and 16.67% at salts and alkaline solutions, respectively. This finding conforms with the results of the bond strength and the previous literature as there is a correlation between material degradation and decrease in bond strength^26^.

### Analytical study

Analytical study was performed to obtain an equation that describes the relationship between the bond strength and the concrete compressive strength for the tested specimens. The statistical analysis resulted in the following proposed equation:

$$f_{b} = 4.3417^{{ \wedge( a1.a2)}} \sqrt {f_{{cu}} }$$

Where:

α_1_ a factor accounts for the exposure environment.

**Table:** 

Condition	α_1_
Control	1
Elevated Water	1.00996355
Salt	1.00563705
Alkaline	1.00103179

α_2_ a factor accounts for the exposure duration.

For Salt solution: α_2_ = -1.39197*10^–6^ t^2^ + 0.000177571 t + 0.995925645.

For Alkaline solution: α_2_ = -1.76959*10^–05^ t^2^ + 0.000672798 t + 0.99574233.

t: Conditioning duration (days); 30 ≤ t ≤  90.

**Fig. 14:**
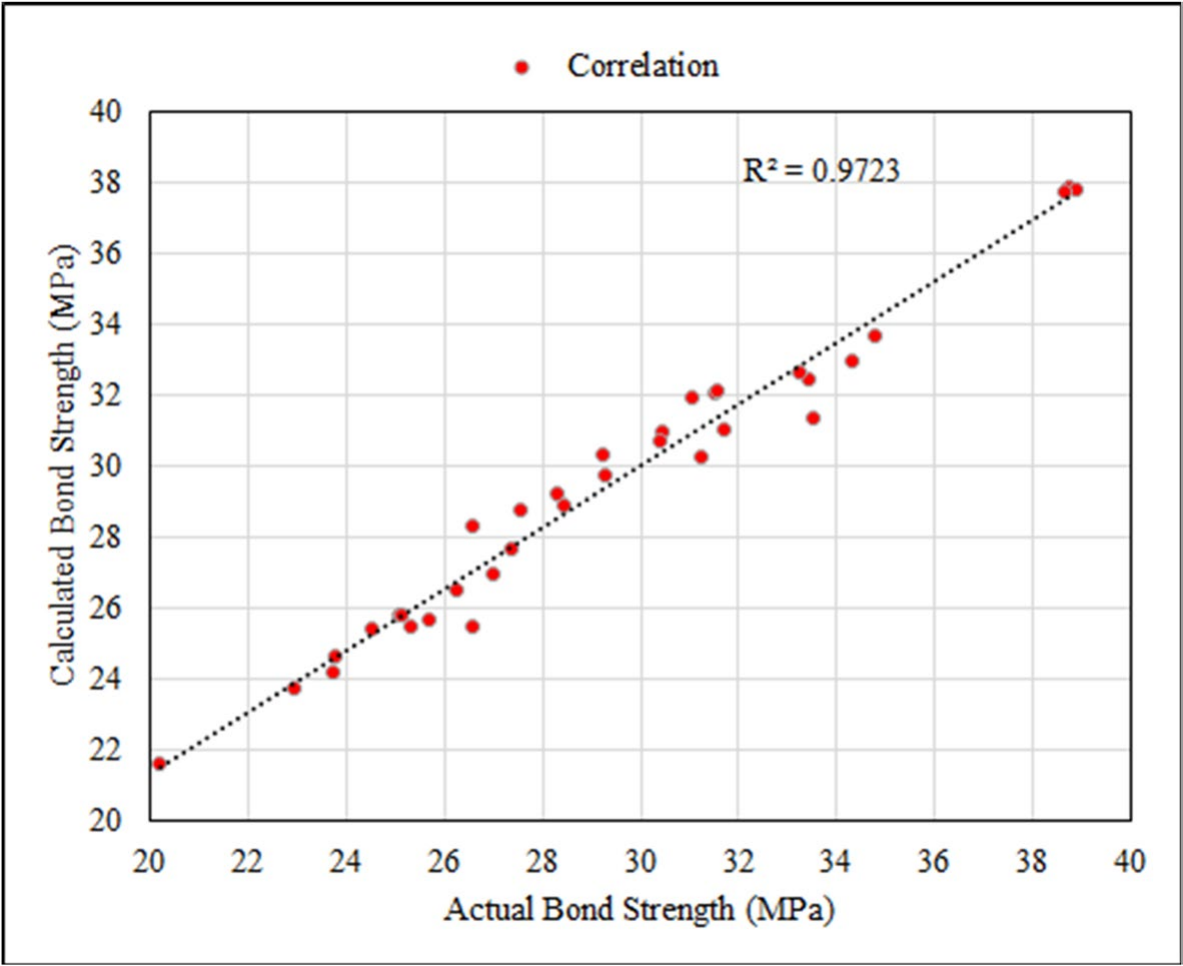
Correlation between the actual and calculated bond strength.

The proposed equation achieved a good correlation between the actual values as shown in Fig. 14.

## Conclusions and recommendations

Seventy-two ribbed BFRP specimens were tested under direct tensile pull-out tests. The following conclusions were extracted from this study:

- The CCS has a governing role on the bond strength durability as the lower the compressive strength the greater the bond strength loss (17.29% for 25 MPa, 12.74% for 45 MPa, and 8.72% for 60 MPa).
- The adhesion to concrete shall be enhanced by swelling of the BFRP bar at the early stages of conditioning. However, in the case of continuous conditioning, these stresses will reduce rapidly.
- The enhancement in bond due to swelling of the bar depends on the absorption properties of the BFRP bar, which depend on the manufacturing quality, and the permeability of the concrete. The permeability of concrete controls the rate of aggressive solution diffusion, thus special attention should be paid to it.
- The exposure to the alkaline solution “simulating the concrete environment” or to the salt solution “simulating the ocean water” had a negligible effect on the bond strength-end slip relationships.
- The governing cause for failure for both control and conditioned specimens was the degradation of BFRP bars, the failure was due to interlaminar shear between the BFRP layers rather than the shear stresses at the interface between concrete and BFRP bar.
- The relation between the bond strength and the compressive strength for the studied BFRP bars can be described through this equation $f_{b} = 4.3417^{{( \wedge a1.a2)}} \sqrt {f_{{cu}} }$.

This study aims to increase the potential of using BFRP bars in constructions through providing a better understanding of the BFRP bars’ behavior. Further research should be devoted to investigating the effect of ultra-high-strength concrete on the bond durability, and the behavior of large-scale specimens.

The experimental study presented is limited to BFRP bars used in this study and should not be extended to other BFRP bar types.

## Data Availability

The datasets used and/or analyzed during the current study are available from the corresponding author upon reasonable request.
